# La Grippe

**Published:** 1891-02

**Authors:** E. Van Goidtsnoven

**Affiliations:** Atlanta, Ga.


### LA GRIPPE.

BY E. VAN GOIDTSNOVEN, M. D., ATLANTA, GA.

If we are to believe the Teutonic School and its many well drilled disciples in this country, it will appear that the germ theory of diseases, is but in its nascent state, and was born only a short time ago of the brain of some German Esculapius. The fact is our press both lay and medical, and the public mind in general can hardly occupy themselves with germs without associating them with Germany.

No political revolution was ever accomplished without blood-shed.

No scientific discovery was ever proclaimed without toil, sacrifice, nay persecution.

Well, I think it is conceded this child had no mother, and it is "a wise kid who can tell its own father!" In looking over the records of claimed paternity, I find the name of one who more than fifty years ago proclaimed to the world this germ theory. His life was one of continuous hardship and struggles in its defense. France, his native land, laughed at him and Germany scorned him. Quantum nentati ab illis! How they have changed since. It can be said of him that he died in the harness, a martyr to the cause!

The bacteriologist who has not heard of Raspail is unworthy of the name and of his profession.

You ask me about Grippe and its trreatment.

Raspail, half a century ago, defined it as the effect of the invasion of a parasite who, impinging upon the isthmus of the larynx and its mucous membrane, determines, through the infiltration of its virus, engorgement of the lympathics and of the muscles of the neck, back and chest, producing as it were, a sort of pathological hood and jacket of conjestion and suffering.

My treatment is as follows:

℞ Antipyrine,

Quiniæ salicylatis, aa ʒ ss. M.

Tere optime et in capsulas. No. xii. Divide. Sig. One capsule every 3 hours. Alternating with:

℞ Ammonii muriatis, ʒ i.

Codeiae, gr. ii. M.

Tere optime et in capsulas No. xii. Divide. Sig. One capsule every three hours.

The treatment should be prescribed in such a way that medicine be administered every hour and a half, strictly observing the rules of alternation.

La Grippe has come to Atlanta to stay, is spreading, and likely to do so for some time. "It's an ill wind which blows nobody any good."

